# Supplementary figures and images for: Computational Modeling of the Chlamydial Developmental Cycle Reveals a Potential Role for Asymmetric Division
Source: mSystems. 2023 Mar 16;8(2):e00053-23. doi: 10.1128/msystems.00053-23 (PMC10134819; doi:10.1128/msystems.00053-23)

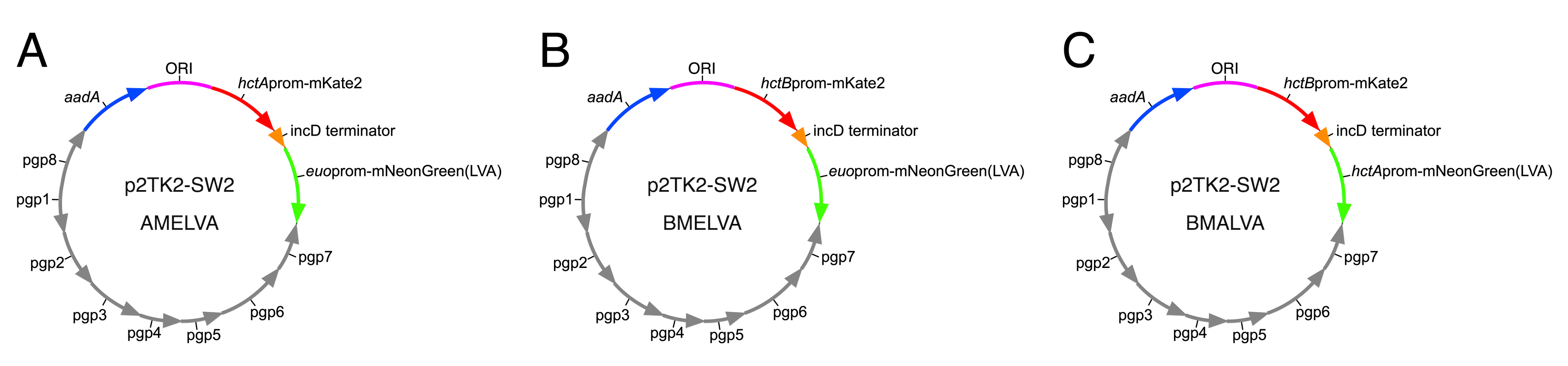

Supplement: FIG S1 [file msystems.00053-23-s0002.tif]

**A**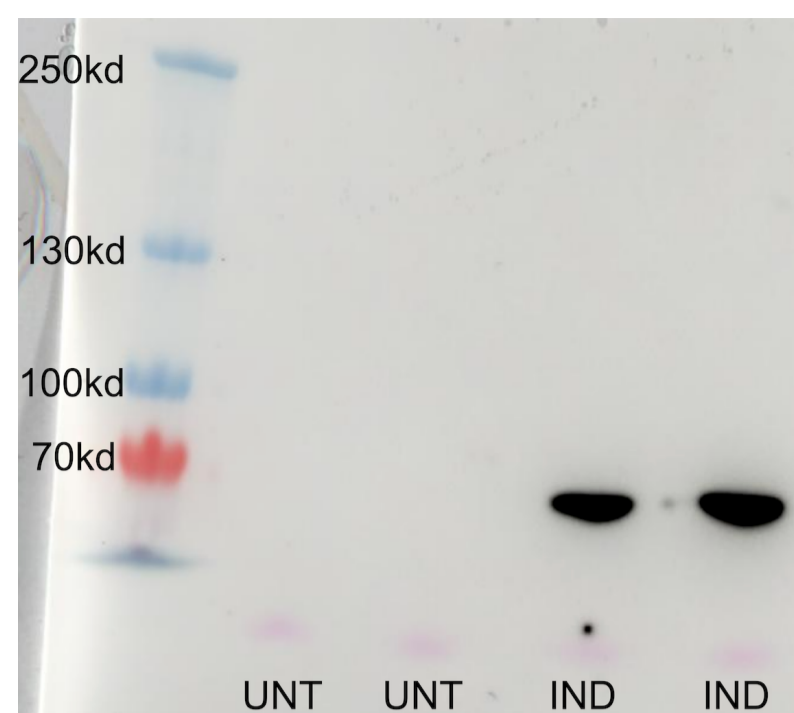**B**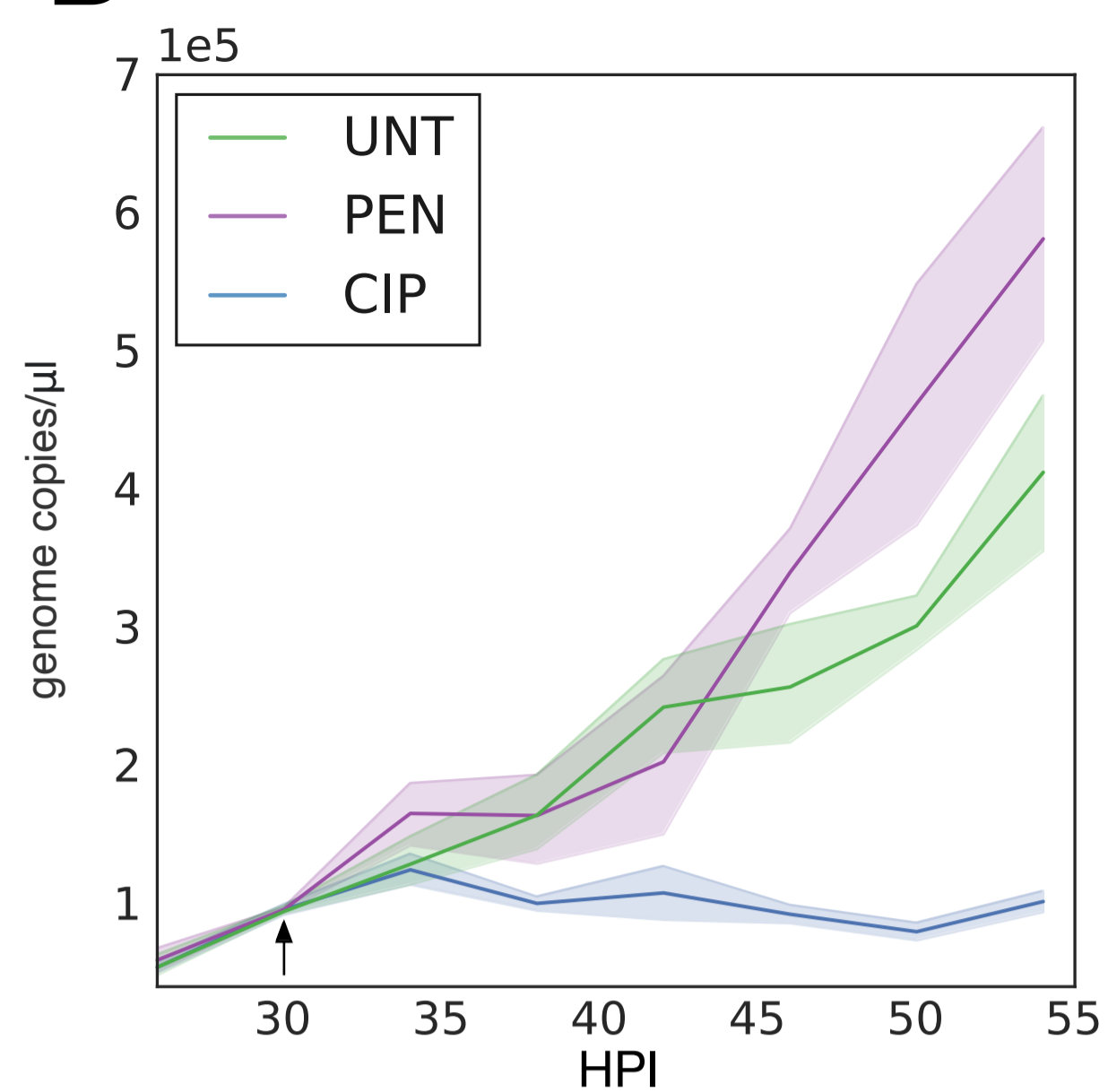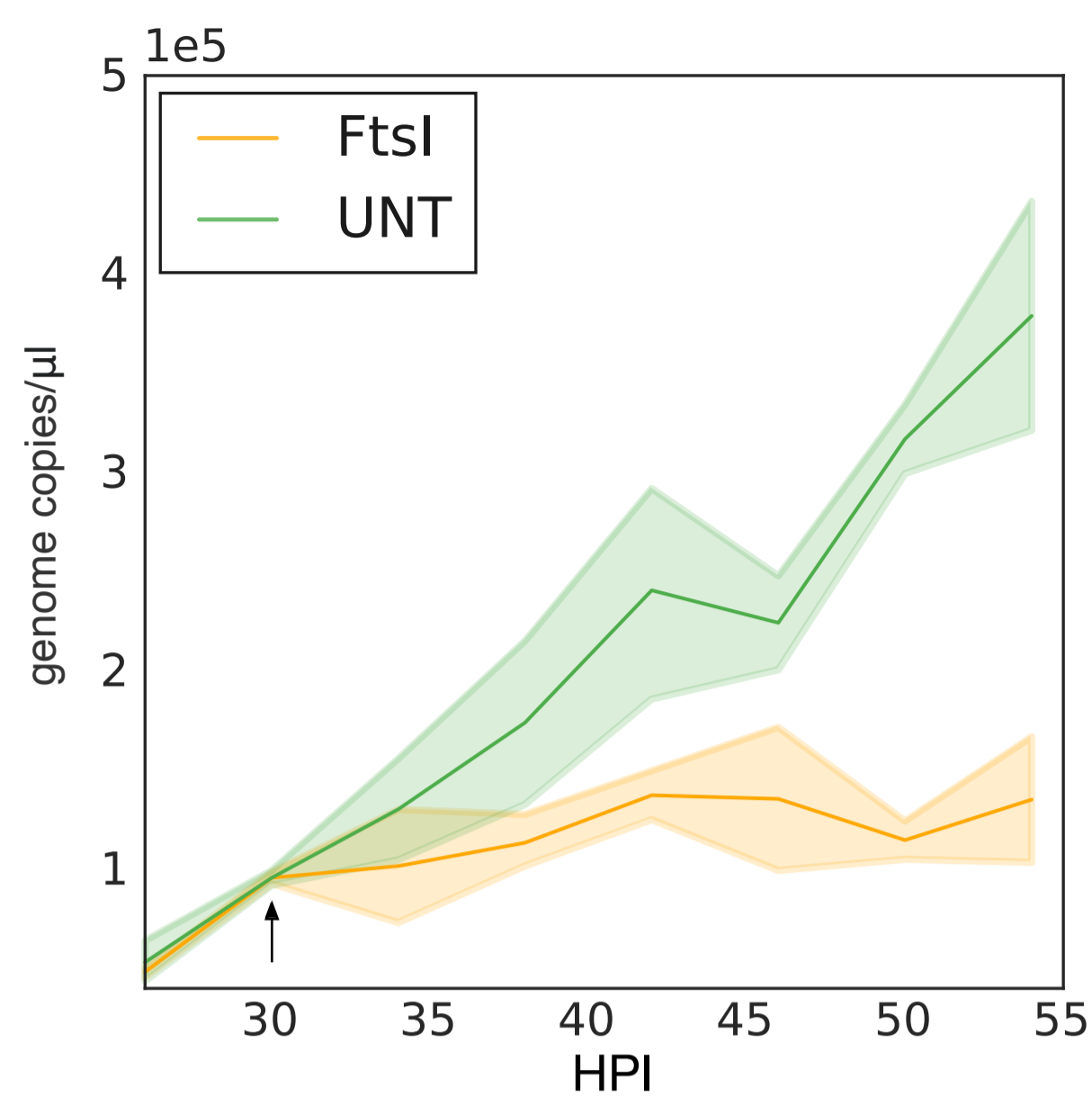**C**

20hpi

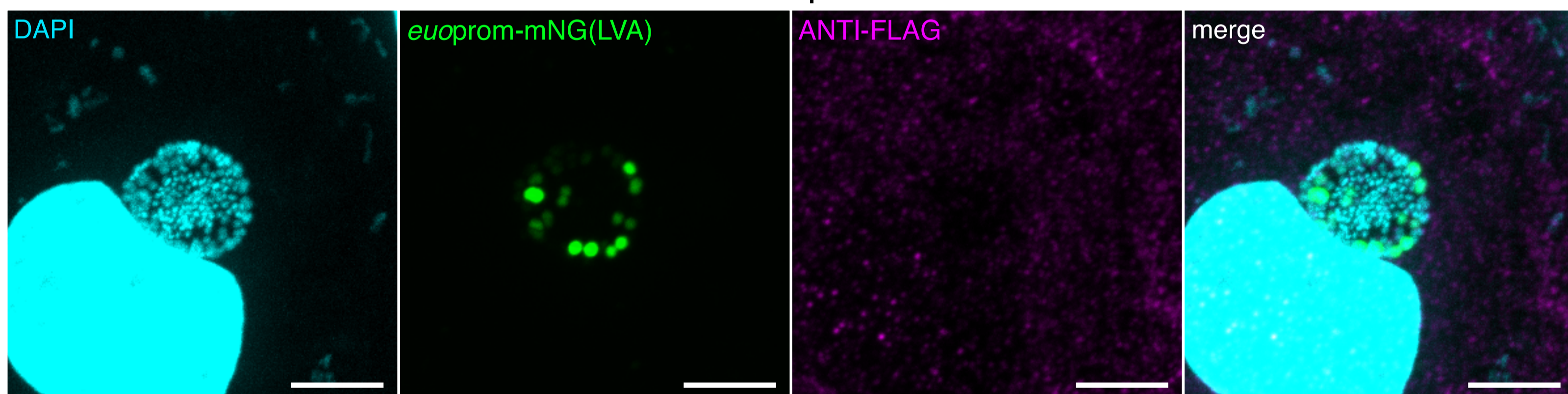

30hpi

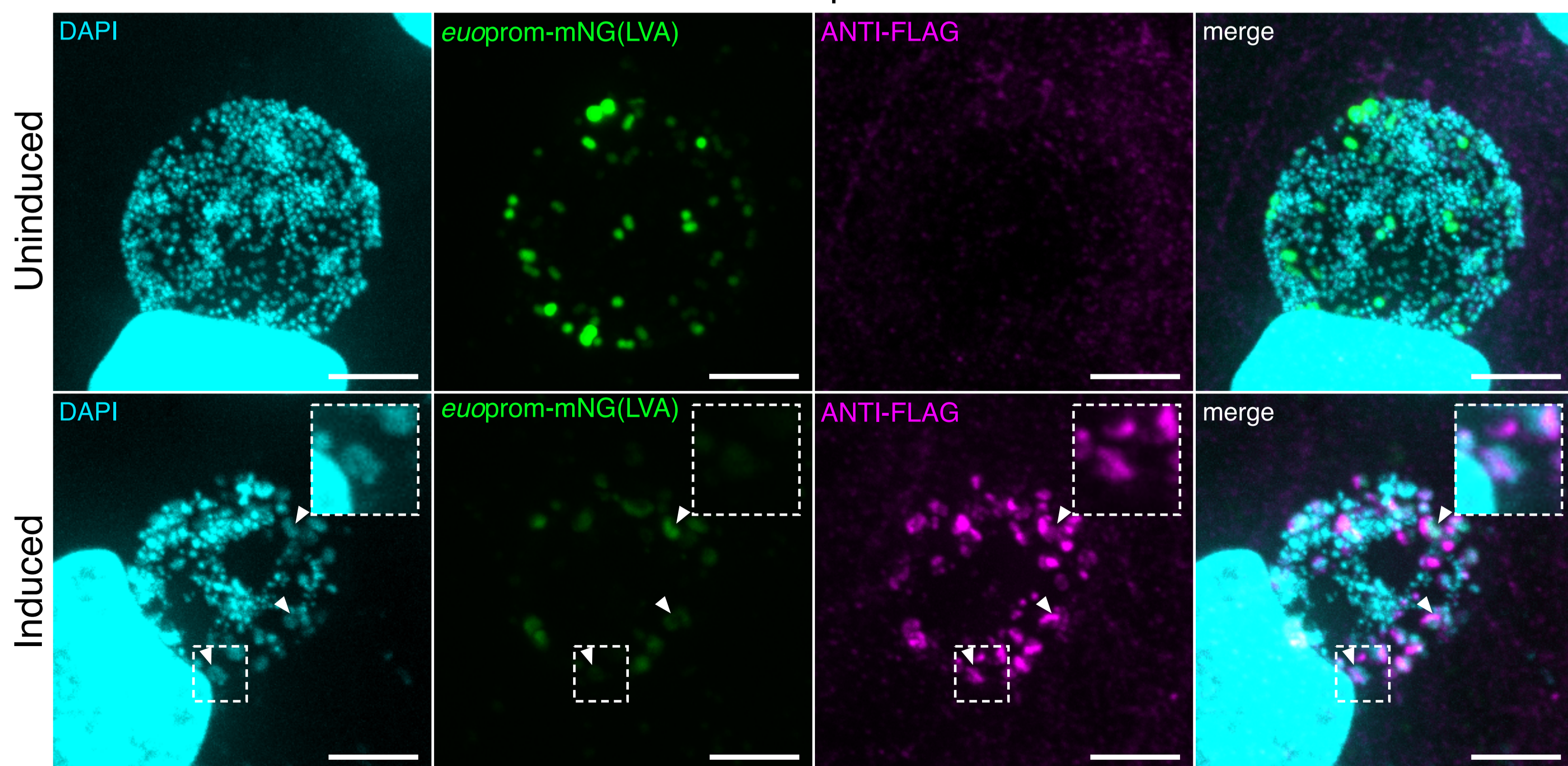

Supplement: FIG S2 [file msystems.00053-23-s0003.pdf]

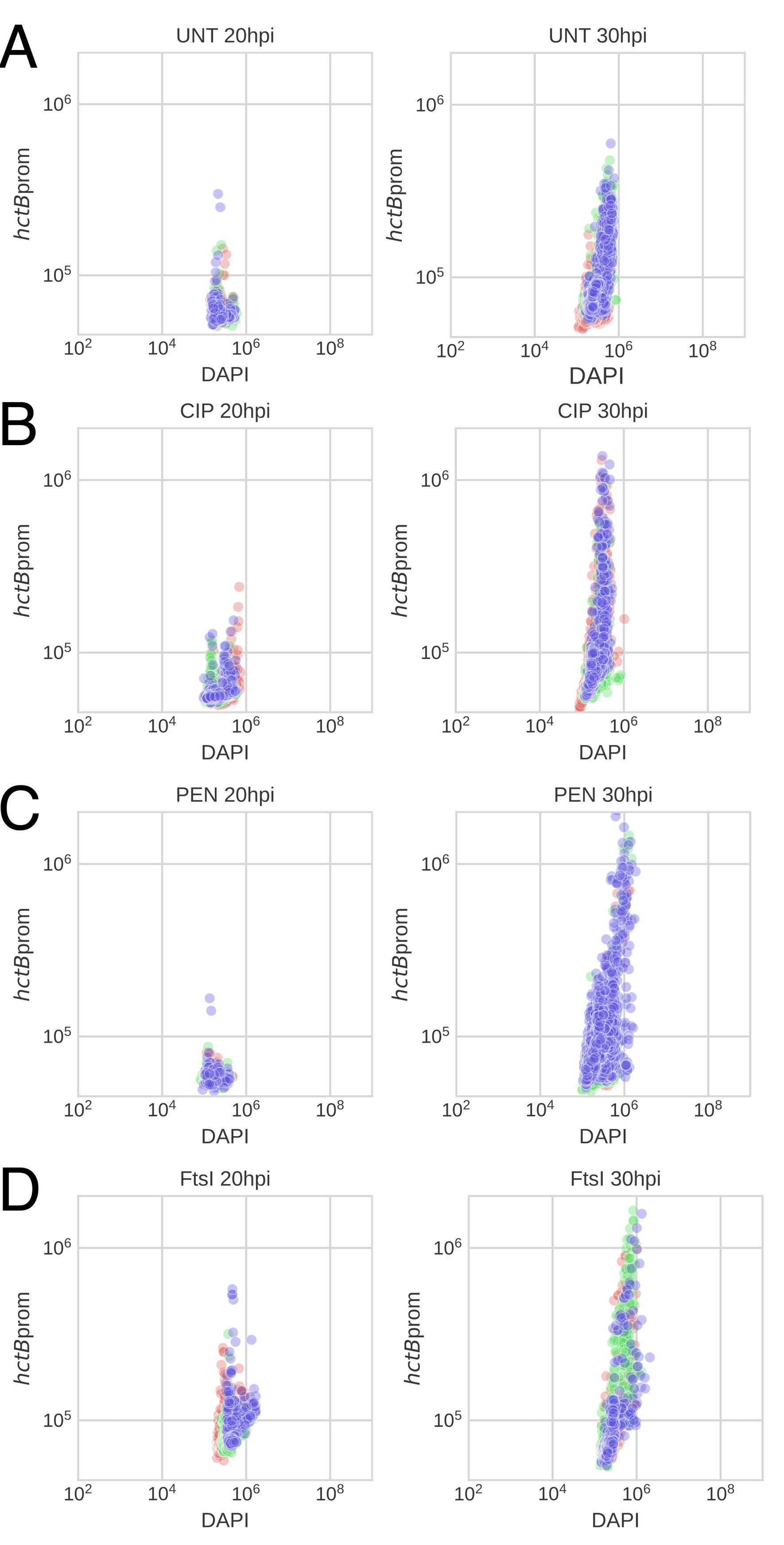

Supplement: FIG S3 [file msystems.00053-23-s0004.tif]

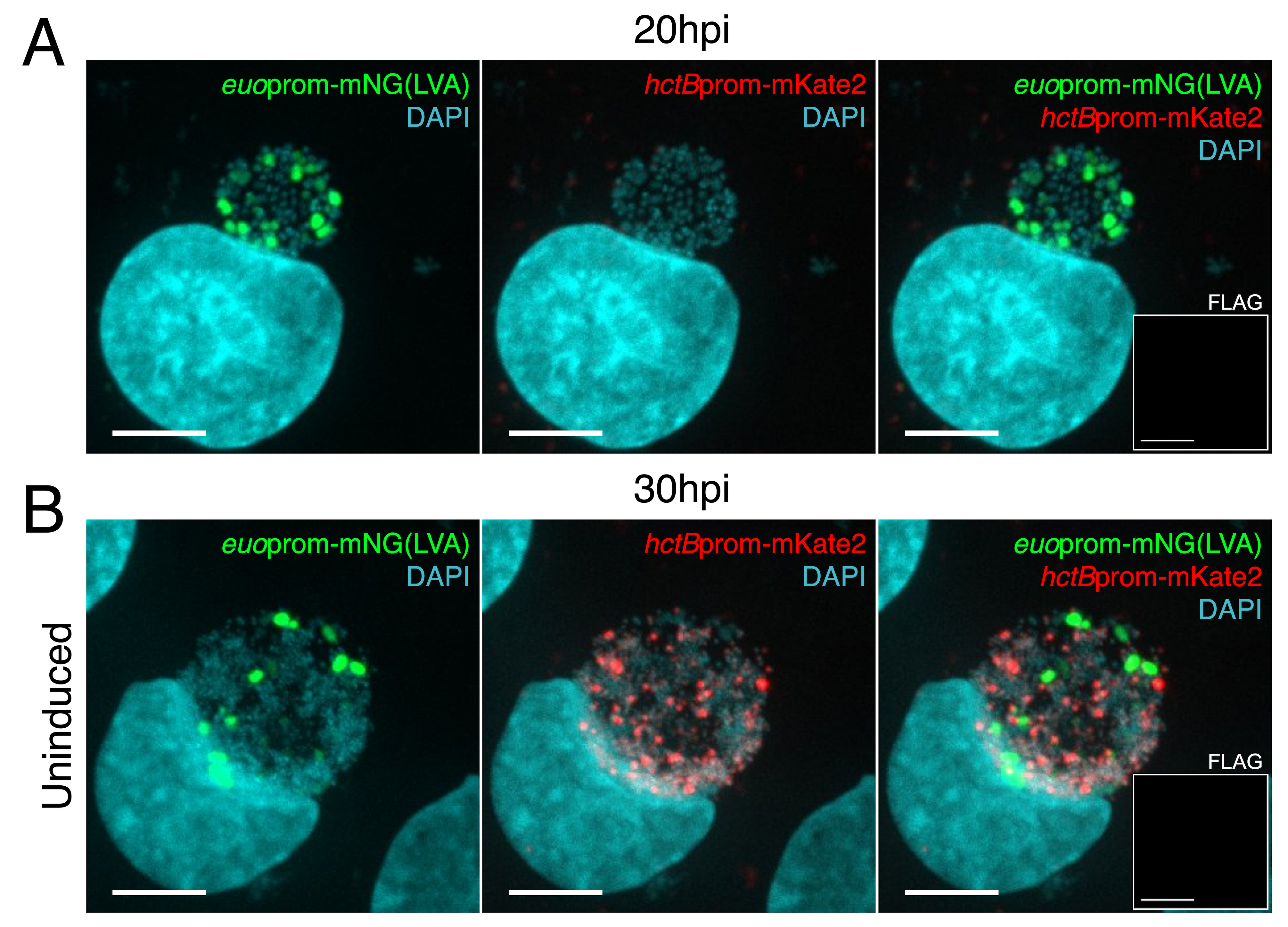

Supplement: FIG S4 [file msystems.00053-23-s0005.tif]
