# Supplementary material for: Computational Modeling of the Chlamydial Developmental Cycle Reveals a Potential Role for Asymmetric Division
Source: mSystems. 2023 Mar 16;8(2):e00053-23. doi: 10.1128/msystems.00053-23 (PMC10134819; doi:10.1128/msystems.00053-23)
Supplement: TABLE S1 [file msystems.00053-23-s0006.docx]

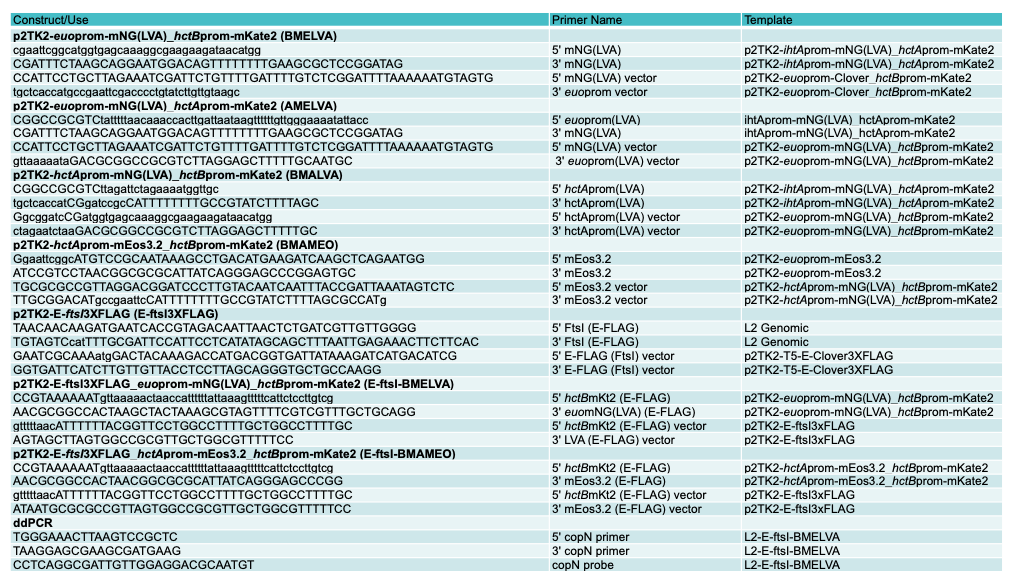


**Table S1.** List of primers used in the construction of the plasmids and ddPCR experiments used in this study.
